# P4CN-YOLOv5s: a passion fruit pests detection method based on lightweight-improved YOLOv5s

**DOI:** 10.3389/fpls.2025.1612642

**Published:** 2025-06-19

**Authors:** Zhiping Tan, Dapeng Ye, Jiancong Wang, Wenxiang Wang

**Affiliations:** ^1^ College of Mechanical and Electrical Engineering, Fujian Agriculture and Forestry University, Fuzhou, China; ^2^ College of Electronics and Information, Guangdong Polytechnic Normal University, Guangzhou, China; ^3^ College of Information Engineering, Gannan University of Science and Technology, Ganzhou, China

**Keywords:** passion fruit pests detection, lightweight deep learning algorithm, YOLOv5S, attention module, pests detection

## Abstract

Passion fruit pests are characterized by their high species diversity, small physical size, and dense populations. Traditional algorithms often face challenges in achieving high detection accuracy and efficiency when addressing the complex task of detecting densely distributed small objects. To address this issue, this paper proposed an enhanced lightweight and efficient deep learning model, which is developed based on YOLOv5s, consists of the PLDIoU, four CBAM modules, and one newAnchors, termed P4CN-YOLOv5s, for detecting passion fruit pests. In P4CN-YOLOv5s, the Mosaic-9 and Mixup algorithms are initially used for data augmentation to augment the training dataset and enhance data complexity. Secondly, after analyzing the image set characteristics to be detected in this research, the point-line distance bounding box loss function is utilized to calculate the coordinate distance of the prediction box and target box, and aimed at improving detection speed. Subsequently, a convolutional block attention module (CBAM) and optimized anchor boxes are employed to reduce the false detection rate of the model. Finally, a dataset consisting of 6,000 images of passion fruit pests is used to evaluate the performance of the proposed model. The experimental data analysis reveals that the proposed P4CN-YOLOv5s model achieves superior performance, with an accuracy of 96.99%, an F1-score of 93.99%, and a mean detection time of 7.2 milliseconds. When compared to other widely used target detection models, including SSD, Faster R-CNN, YOLOv3, YOLOv4, YOLOv5, P4C-YOLOv5s, and YOLOv7 on the same dataset, the P4CN-YOLOv5s model demonstrates distinct advantages, such as a low false positive rate and high detection efficiency. Therefore, the proposed model proves to be more effective for detecting passion fruit pests in natural orchard environments.

## 1 Introduction

Passion fruit, which has a significant economic value, often suffers from pest infestation during its growth, leading to a decline in quality and yield. It causes losses not only to the farmers but also to the agricultural economy (Pereira et al., 2023).

The prerequisite for pest control is the timely and accurate detection of pests. Real-time pest detection in crops with the application of scientific methods is a vital tool in the current cultivation and management of crops. Initially, the researchers used novel techniques for pest detection with positive achievements. These novel techniques were effective in reducing labor costs and increasing detection rates. Moreover, the novel techniques also conserve resources to limit the negative impact on the environment. However, these methods are ineffective in the actual field environment (Cai et al., 2024).

The advancement of techniques in deep learning has achieved a wide range of successful applications of models in computer vision (CV) such as the convolutional neural network (CNN) and the transformer. Applications such as traffic detection (Mahrez et al., 2021), face recognition (Wang and Guo, 2021), and pedestrian detection (Li et al., 2018) are included. Crop pest and disease monitoring has gradually developed from traditional manual monitoring to automation, informationization, and intelligence (Juan et al., 2023). Deep learning technology provides new solutions and opportunities for agriculture by utilizing big data and powerful computing capabilities. Researchers have already used CNN and deep learning techniques in agriculture to develop intelligent agriculture. Intelligent agriculture can help agricultural workers to improve productivity, reduce resource waste, and promote sustainable agriculture (Xiaodong et al., 2021) (Kartikeyan and Shrivastava, 2021). A popular intelligent method currently used in pest detection is target detection (Ding and Taylor, 2016; Qin et al., 2023). Being an image processing technique, the target detection aims at identifying and localizing specific objects from images or video streams. Target detection provides fundamental support for computer vision applications, where two-stage algorithms and single-stage algorithms are its mainstream algorithms.

Two-stage algorithms are RCNN series algorithms (Girshick et al., 2013; Girshick, 2015; Ren et al., 2017). These algorithmic models have two main stages in detection. The first step is to generate candidate regions on the to-be-detected image. The second step is the objects are detected based on candidate regions with CNN. A three-pest detection method for lychee with an accuracy of more than 95% that is based on deep learning has been proposed by Jin Y. et al (Jin et al., 2021). A multi-class pest detection method called PestNet is proposed by Liu et al (Liu et al., 2019), which had an average accuracy of 75.46% on the Multi-Class Pest Dataset 2018 (MPD2018). The mini-CNN structure proposed by (Rahman et al., 2020). is a two-stage algorithm. This structure allows the model to maintain 93.3% accuracy while reducing its size by 99%. It provides timely crop disease detection for under-resourced devices.

The single-stage algorithms are Single Shot MultiBox Detector (SSD) algorithm (Liu et al., 2016) and You Only Look Once (YOLO) series algorithms (Jocher et al., 2020; Redmon et al., 2016; Redmon and Farhadi, 2017; Redmon and Farhadi, 2018; Bochkovskiy et al., 2020). These methods do not generate candidate regions at the time of detection but solve the problem of localizing and classifying the target in a regression approach. It means that the model can get the final detection result directly after only one stage. In 2022, (Zhang et al., 2022). proposed a lightweight model, called AgriPest-YOLO model, with better accuracy than the classical detection model, and it can detect 24 categories of pests with a mean precision of 71.3%. (Hu et al., 2023). proposed the YOLO-GBS model by merging the global context (GC) attention module, which can recognize the insect dataset of Crambidae in complex backgrounds with mAP of 79.8%. Zhang and Ma et al (Zhang et al., 2022). proposed a modified YOLOX model that adds efficient channel attention (ECA), replaces the activation function with the Swish function, and works with the Focal Loss function. These modifications improved the YOLOX model’s performance in detecting cotton pests and diseases, and its average accuracy reached 94.60%.

Although the two-stage algorithm performs well in localization and classification, it requires two stages to output the results, which is time-consuming and cannot meet the requirement of immediacy. By contrast, the single-stage algorithm directly outputs the detection rate and the positional coordinates of the target through a single detection, which is faster. However, some of the single-stage algorithms are also flawed. SSD (Liu et al., 2016) is weak in recognizing small objects. YOLOv1 (Redmon et al., 2016) localizes prediction boxes and classifies them directly at the output layer, but it recognizes dense objects and small objects very poorly with low accuracy. Though YOLOv2 (Redmon and Farhadi, 2017) uses high-resolution images to build a classification network, improving detection speed, accuracy, and classification number, its prediction of overlapping or small objects is poor. YOLOv3 (Redmon and Farhadi, 2018) speeds up the computation. It can be used to quickly identify objects under complex situations such as small objects and similar backgrounds. On the contrary, its training speed is slow and its generalization is poor. Although YOLOv4 (Bochkovskiy et al., 2020) improves the model outcome by balancing detection accuracy and speed, it has a high false detection rate. YOLOv5 (Jocher et al., 2020), proposed by Jocher et al. in 2020, performs well in target detection applications with low false-detection rate and high performance, and its pre-trained model is very small, only about 10% of the YOLOv4 model. It is also applicable to various application scenarios such as multi-image, video and real-time monitoring. YOLOv5 has small (s), medium (m), large (l), and extra-large (x) model structures, namely YOLOv5s, YOLOv5m, YOLOv5l, and YOLOv5x. It improves model robustness and inference speed on the input layer with Mosaic data augmentation, adaptive adjustment of anchor box, and adaptive image scaling. Its Backbone layer includes the Cross Stage Partial Network (CSP) and Spatial Pyramid Pooling-Fast (SPPF) module. The CSP network structure optimizes the CNNs in the model, which not only further improves the capability of learning, but also maintains the accuracy. The SPPF network structure enables models to simplify calculations, reduce training time and optimize training results without loss of accuracy. The Feature Pyramid Networks (FPN) (Lin et al., 2017) and Path Aggregation Network (PAN) (Liu et al., 2018) in the neck layer fuse the feature maps from different stages to further improve the detection results.

For the above reasons, the YOLOv5s is suitable for passion fruit pest detection with its lightweight and deployable features. The recognition methods described above have low accuracy or are mostly limited to specific images and regions. None of them are suitable for the detection and identification of passion fruit pests. To optimize the identification and localization of passion fruit pests, a P4CN-YOLOv5s model based on our previous research (P4C-YOLOv5s) (Zhang et al., 2018; Selvaraju et al., 2017) is proposed. The P4CN-YOLOv5s model offers a lower false detection rate and shorter detection time, making the following innovative improvements in this study.

1. Dataset Reprocessing and Anchor Boxes Optimization: In this study, we introduce a novel approach to dataset enhancement by collecting real pest data and applying the Mosaic-9 and Mixup algorithms at the input layer of the model. This reprocessing technique not only increases data complexity and the number of small objects but also significantly improves the model’s robustness and prediction performance. Additionally, we optimize anchor box values by employing the K-means clustering algorithm, which enhances the model’s accuracy.
2. Neck Layer Optimization with CBAM: We propose an innovative enhancement to the neck network by incorporating the Convolutional Block Attention Module (CBAM). This module adapts the convolutional neural network to focus more effectively on the target by increasing attention to relevant features. The CBAM also allows for the decomposition of original features into more refined representations, offering the model richer contextual information and enabling more accurate data understanding and categorization.
3. Introduction of the PLDIoU Loss Function: A novel bounding box regression loss function, Point Line Distance Intersection over Union (PLDIoU), is introduced to improve localization accuracy. PLDIoU reduces redundant computations and accelerates the localization process by effectively representing the distance between predicted and target box coordinates, contributing to more efficient and accurate bounding box predictions.

## 2 Data collection and optimization

### 2.1 Data collection

The passion fruit pest dataset used in this study consists of a combination of a self-collected dataset and a publicly available dataset from PaddlePaddle. After filtering, a total of 2,811 high-quality images were retained. To enhance the dataset, we expanded it to 6,000 images by randomly sampling from the original set and applying selected data augmentation techniques. A total of 12 data augmentation methods were employed in this process, including size scaling, grayscale conversion, center cropping, random cropping, random cropping with scaling, edge padding, random rotation, horizontal flipping, vertical flipping, color dithering, and affine transformation. The dataset was initially divided into 10 equal parts, with 8 parts allocated for training and validation, and the remaining 2 parts used for testing. Subsequently, the training-validation set was further divided into 10 parts, where 7 parts were used for training and 3 parts for validation. As a result, the training set contained 3,360 images, the validation set contained 1,440 images, and the test set consisted of 1,200 images.

A total of 12 pests are included in the dataset, which are Bactrocera dorsalis (Bd), elater, Epicauta ruficeps (Er), Halyomorpha halys (Hh), Prodenia litura-Adult (PlA), Prodenia litura-Larva (PlL), Red spider (Rs), Scarab beetle (Sb), Sympiezomia citre (Sc), slug, snail and thrips. There are approximately 500 images of each pest. Detailed data of the dataset is presented in **Table 1**.

**Table 1:** The statistics of passion fruit pest dataset.

Labels	Number of collection	Number for data augmentation	Number for training	Number for test
Bd	158	342	272	110
elater	269	231	282	89
Er	279	221	280	109
Hh	111	389	279	117
PlA	163	337	262	105
PlL	169	331	284	87
Rs	198	302	298	95
Sb	366	134	287	90
Sc	279	221	271	100
slug	286	214	269	99
snail	275	225	278	104
thrips	258	242	298	95

LabelImg is a graphical image labeling tool that is often used in data annotation for object detection. Before training, the dataset is labeled with the LabelImg software, and the label information is saved as Extensible Markup Language (XML) files in PASCAL VOC 2007 format. The XML file records the original image information, object name, and object coordinates in detail. Besides, the labeling information of this format file is also supported for the training of YOLO series models, SSD algorithm, and R-CNN series algorithm models. It is convenient for comparison experiments.

**Figure 1:**
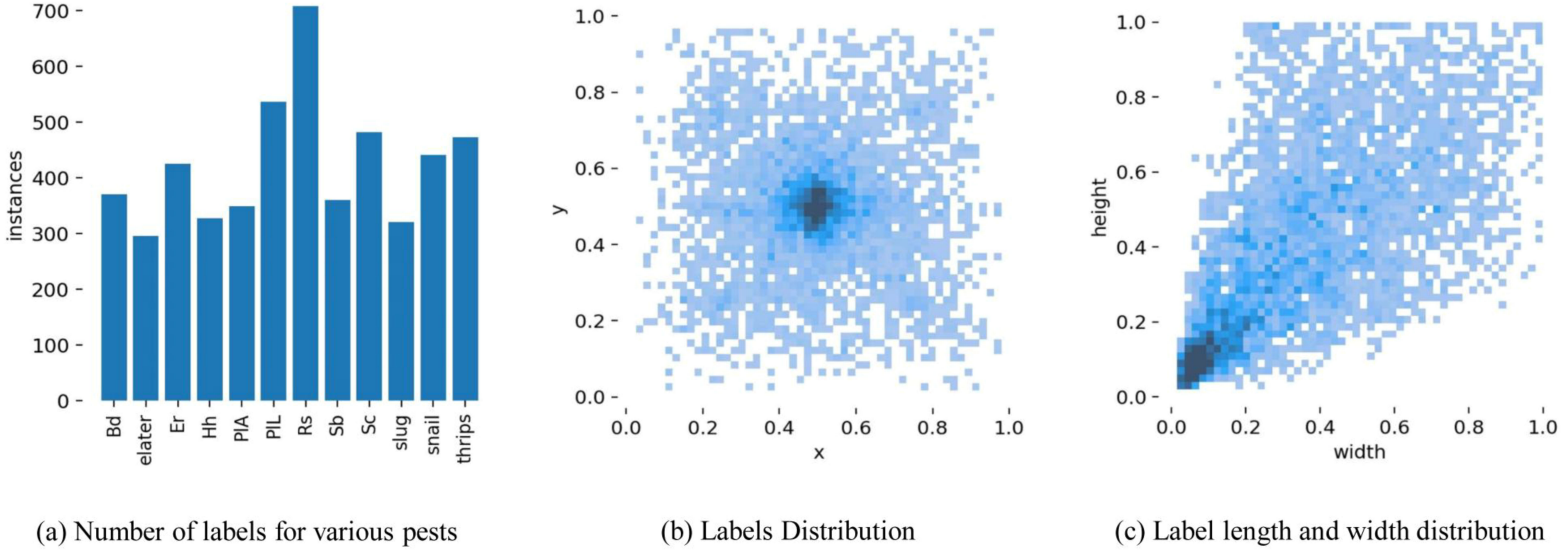
The diagram of the pest labeling data **(a)** number of labels for various pests, **(b)** labels distribution, **(c)** label length and width distribution.

Meanwhile, **Figure 1** provides the corresponding data distribution of the pest labeling data. **Figure 1a** shows the amount of labels for each type of pest. **Figure 1b** shows the coordinate distribution of each label and the labels are mostly concentrated in the middle of the image. **Figure 1c** shows the size share of the label box in the image. It can be seen that the largest proportion is small object pests, indicating that the model should focus on small object pests.

### 2.2 Dataset reprocessing

The Mosaic-9 algorithm and the Mixup algorithm are added to the model input layer to improve data complexity and model robustness and increase the number of object pests.

**Figure 2:**
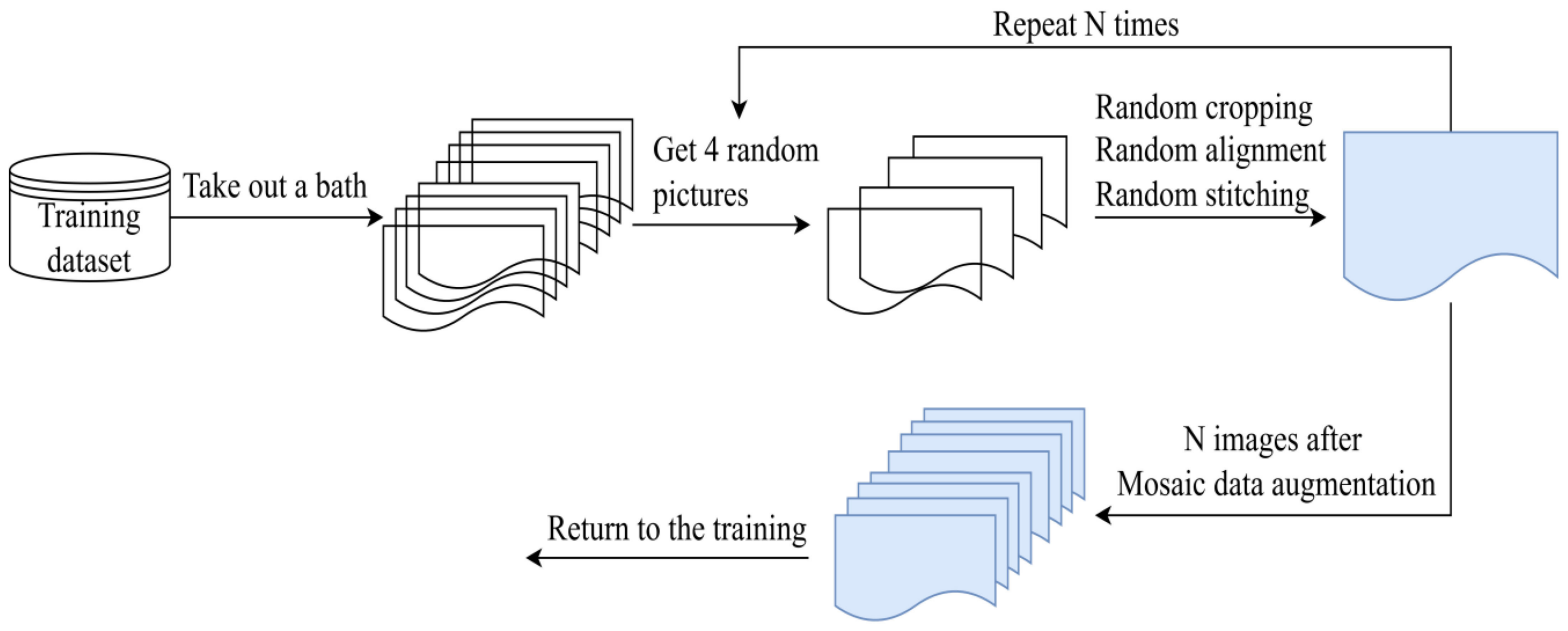
A simple flowchart of the Mosaic data augmentation algorithm.

**Mosaic-9.** The mosaic algorithm helps in scaling up the training data size and increasing the data diversity, thus improving the training results. It has two main key steps. The first is to pick four images from the training dataset. Then all 4 images are randomly cropped with a small part and the cropped images are stitched into a new image wsith a certain ratio. And the Mosaic algorithm will calculate the data of four images when performing the normalization operation. Therefore, the memory consumption of the model is reduced. **Figure 2** presents a simplified demo of the mosaic algorithm. *N* is the batch size.

**Figure 3:**
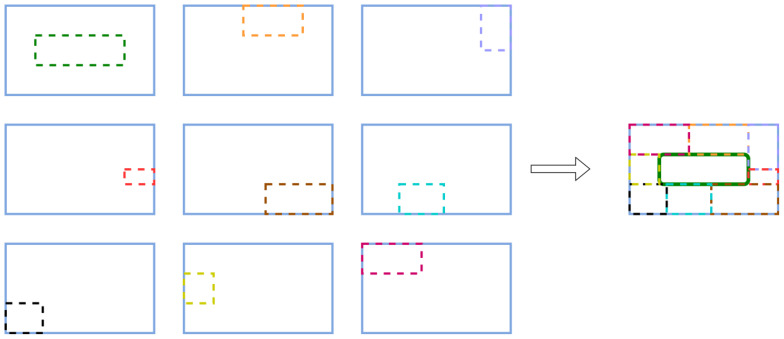
An example diagram of the Mosaic-9 data augmentation algorithm.

We upgrade the Mosaic method from randomly stitching 4 images to randomly stitching 9 images to get the Mosaic-9 online data augmentation algorithm. Compared with the Mosaic algorithm, the Mosaic-9 algorithm makes the dataset more complex and increases the percentage of small targets, which makes the model more stable. **Figure 3** shows an example operation of the Mosaic-9 algorithm.

Unlike the traditional Mosaic algorithm, the Mosaic-9 algorithm selects 9 images randomly and then intercepts portions from the 9 images respectively with specific rules. Eventually, the intercepted 9 images are merged into a new image by the same rules. Repeat this several times to get several new images.

**Mixup**. The Mixup algorithm is used to mix two randomly selected images in a ratio to create new data with labeled information. It can complicate and expand the dataset (Liu et al., 2018).

**Figure 4:**
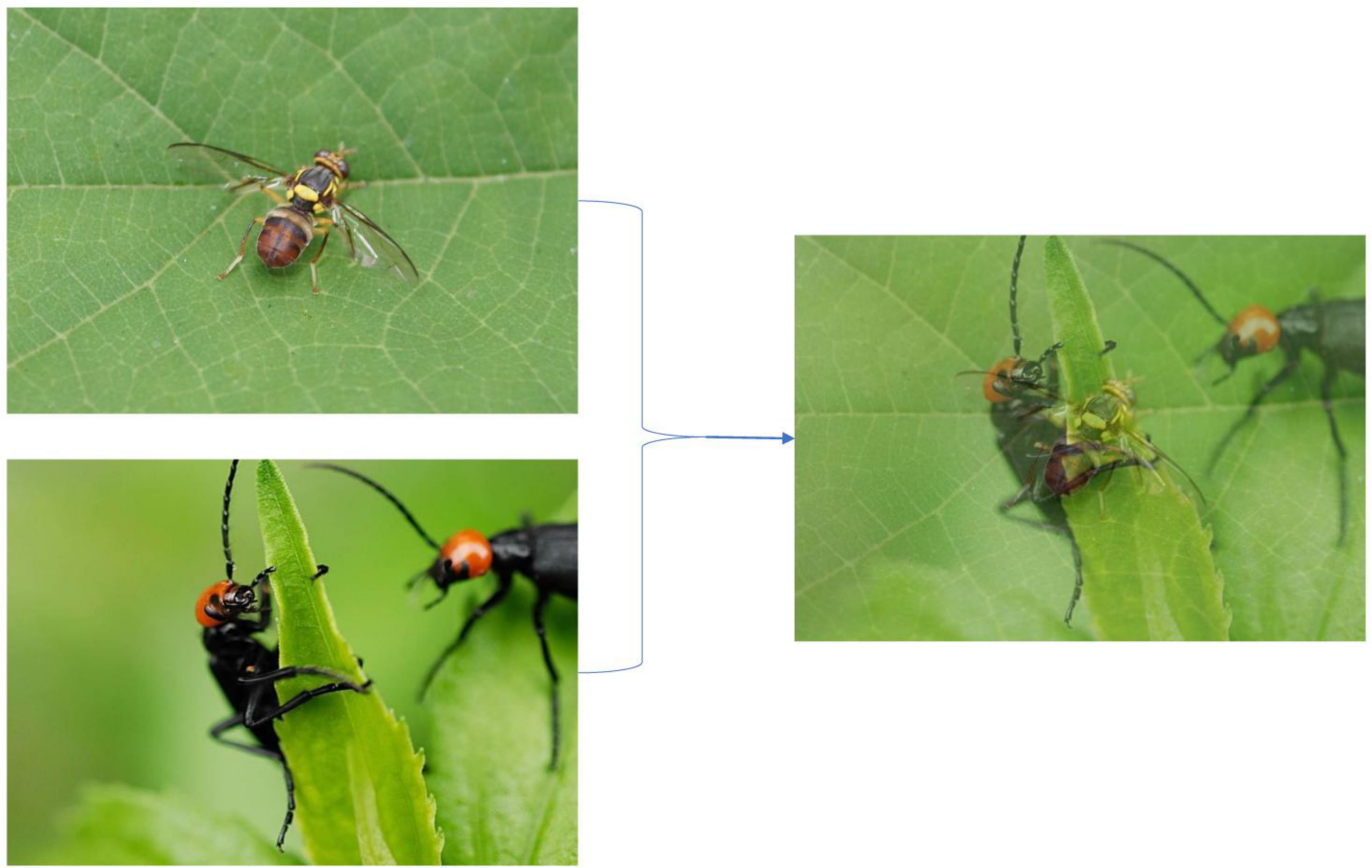
Example of mixup.

**Figure 4** is a simple step of the Mixup algorithm execution. The Mixup algorithm improves model stabilization and prevents overfitting. It is insensitive to noisy samples and improves the model’s ability to learn the hidden regularities behind the data with improved generalization. Its best feature is that it is readily available and has a negligible impact on memory.

### 2.3 Anchor boxes optimization

As an important part of object detection, anchors may vary on different datasets. A suitable anchor can substantially improve the effectiveness of the model. In practical applications, anchors need to be reselected according to specific datasets. During the training process, we found that the function of YOLOv5 to automatically calculate the anchor values did not take effect. To get suitable anchors, we recalculated them with the K-means clustering algorithm. The values of the new anchors (newAnchors) are [(41,43), (92,77), (123,172)], [(210,124), (216,229), (335,192)] and [(255,350), (469,261), (442,405)].

## 3 The proposed method

YOLOv5 continues to undergo updates, and its structural diagrams may vary across different publications. In this study, the YOLOv5 architecture is based on version 6. The authors of YOLOv5 have developed the YOLOv5 family with an emphasis on streamlined and efficient module packaging, resulting in code that is highly readable and easy to implement. The YOLOv5 architecture mainly comprises four components: the input layer (Input), the backbone layer (Backbone), the neck layer (Neck), and the prediction layer (Prediction).

### 3.1 P4CN-YOLOv5s

**Figure 5:**
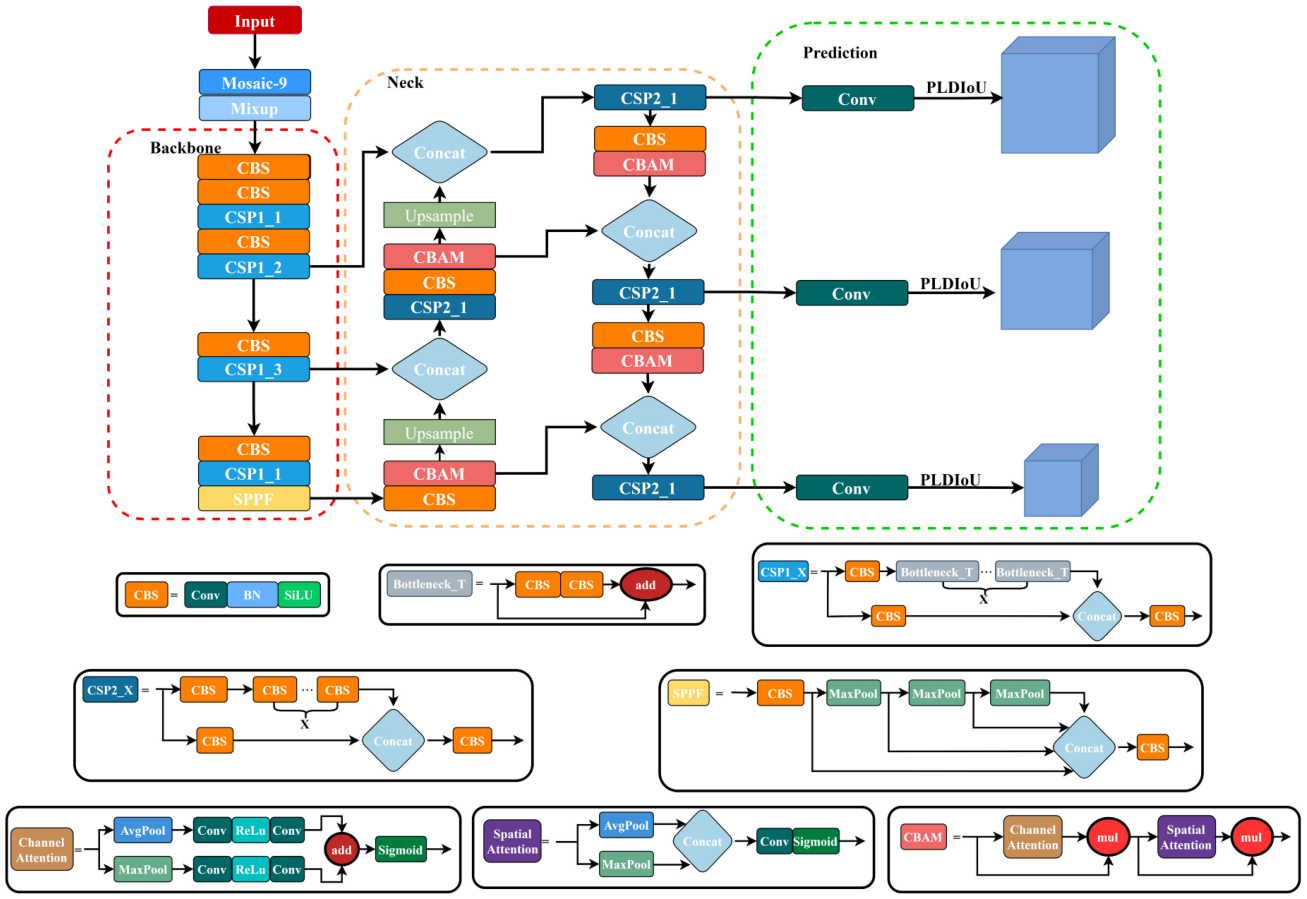
P4CN-YOLOv5s network structure diagram.

Since most passion fruit pests are small and the dataset is not very enough, the traditional YOLOv5 model is generally effective in detecting them. In this paper, the P4CN-YOLOv5s model, based on pyramid pooling for contextual networks is proposed for passion fruit pests detection and identification. As **Figure 5** shows it is the proposed P4CN-YOLOv5s model schematic. The specific design is as follows: Firstly, the training dataset is reprocessed. The input layer is added with the Mosaic-9 algorithm and the Mixup algorithm to enrich training datasets, enhance image complexity and the number of objects, as well as strengthen model robustness and generalization. Secondly, the anchor boxes are optimized. The anchor boxes are readjusted that match the dataset of this study to improve the model performance and localization. Thirdly, the neck network layer is optimized. Four CBAM attention modules are added to the neck layer. This improvement provides the model with the ability to concentrate more on the target object, get its key information and features, and effectively reduce the interference of invalid information. Finally, a new PLDIoU loss function is introduced. Instead of YOLOv5’s original loss function, we propose a PLDIoU loss function to reduce unnecessary computations and speed up the detection. The dataset reprocessing and anchor boxes optimization are described in subsections 2.2 and 2.3. The next subsections 3.2 and 3.3 will focus on neck layer optimization and PLDIoU.

### 3.2 Neck layer optimization

In visual tasks, each image contains regions that attract varying levels of attention, and not all pixels contribute equally to the model’s decision-making. Attention modules help address this by enabling the network to automatically learn a set of weighting factors, which are then applied dynamically to emphasize important regions while suppressing less relevant ones. By integrating attention modules into neural network models, the network can better capture key features, thereby enhancing its focus and overall performance.

Attention mechanisms are typically categorized into three types: channel attention, spatial attention, and hybrid (or mixed) attention modules. The channel attention mechanism assigns importance weights to different feature channels based on learned information, allowing the network to enhance critical features and suppress less informative ones. In contrast, the spatial attention mechanism directs the model’s focus toward specific spatial regions within the image. It uses spatial transformations to re-encode the spatial information while preserving key content, subsequently generating spatial weights to highlight target regions.

**Figure 6:**
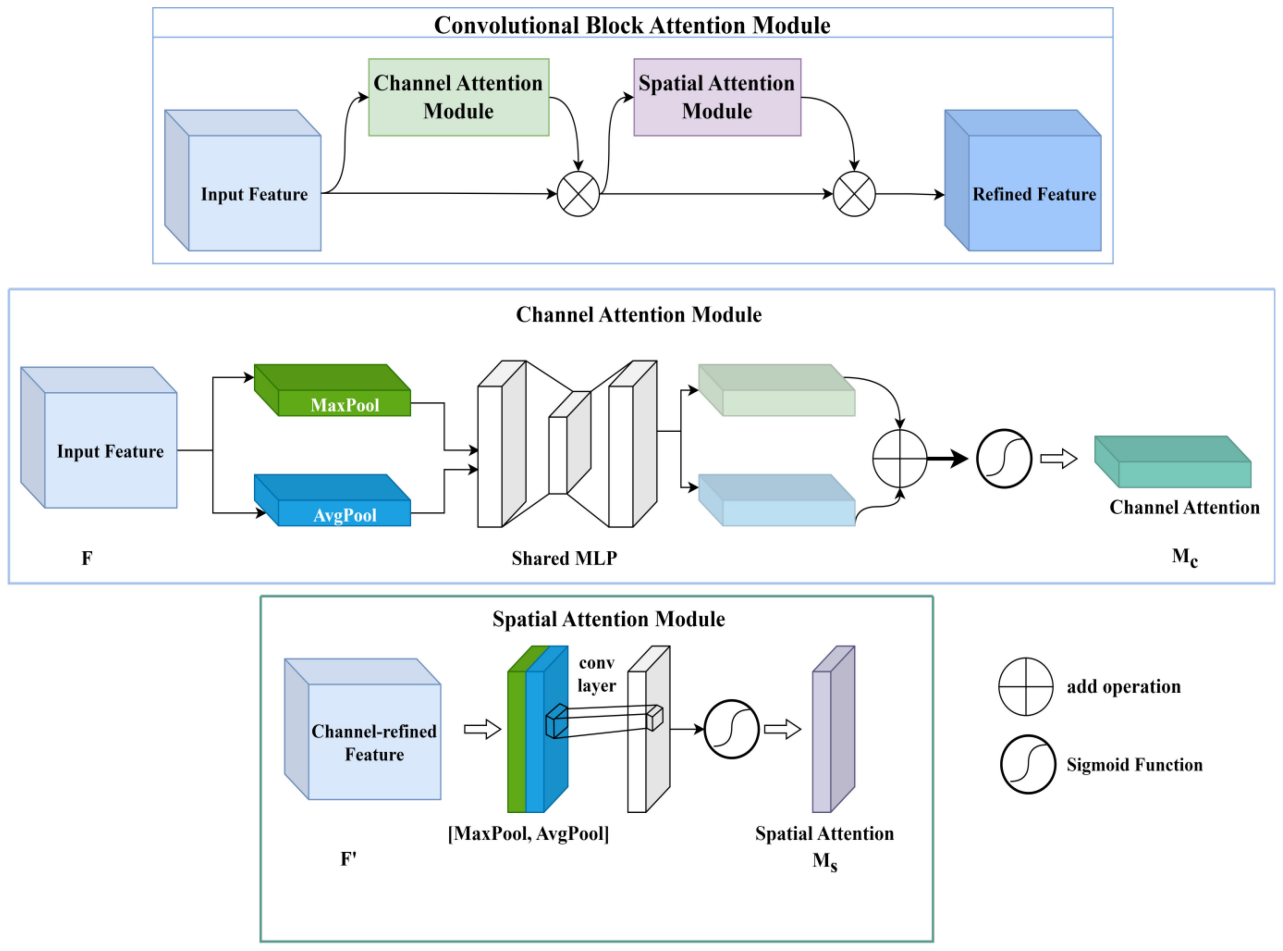
The CBAM module simplified diagram.

However, the channel attention module finds it easy to ignore the information exchange within the space, and the spatial attention module finds it easy to ignore the information exchange between the channels. The mixed attention module is created by the combination of the channel attention module and the spatial attention module with parallel or series connections. It balances both channel and spatial information exchange and has the advantages of both channel attention modules and spatial attention modules. The convolutional block attention module (CBAM) (Woo et al., 2018) is a mixed attention module. Its schematic is shown in **Figure 6**. The CBAM links the channel attention module and the spatial attention module in series to adjust the input feature maps, from which the detailed feature maps are output.

At first, the input feature map $F\in {\mathbb{R}}^{C\times H\times W}$ (C denotes channel, H denotes height, and W denotes width.) is adjusted by the channel attention module in the CBAM module. Two one-dimensional vectors are created after *F* is pooled by max-pooling and average-pooling. After that, the two vectors are passed to a multilayer perceptron (MLP). The MLP outputs a one-dimensional channel attention graph $M_{c}\in {\mathbb{R}}^{C\times 1\times 1}$ . $M_{c}$ is multiplied with the initial input *F* to output the feature map *F’*.

After the channel attention module outputs the *F’*, the spatial attention module will immediately adjust *F’*. In the spatial attention module, *F’* will be pooled for the first time. The pooling sequence is max pooling first and average pooling last. Two 2-dimensional vectors are output after *F’* is pooled. These two vectors will be input to a standard convolutional layer for convolutional operation, which outputs the 2D spatial attention $M_{s}\in {\mathbb{R}}^{1\times H\times W}$ . $M_{s}$ and the original input *F’* are multiplied to output the final refined feature map *F”*.

The formulas for the CBAM output of the refined feature map are given below (Equations 1–3).


(1) $$\begin{array}{l} F^{\prime }=M_{c}(F)⊗F,\\ F^{\prime \prime }=M_{s}(F^{\prime })⊗F^{\prime } \end{array}$$

(2) $$\begin{array}{c} M_{c}(F)=\sigma (MLP(AvgPool(F))+MLP(MaxPool(F)))\\ =\sigma (W_{1}(W_{0}(F_{avg}^{c}))+W_{1}(W_{0}(F_{\max}^{c}))) \end{array}$$

(3) $$\begin{array}{c} M_{s}=\sigma (f^{7\times 7}([AvgPool(F);MaxPool(F)]))\\ =\sigma (f^{7\times 7}([F_{avg}^{s};F_{\max}^{s}])) \end{array}$$

where *c* denotes the channel attention module. *s* denotes the spatial attention module. ⊗ denotes element-wise multiplication. *σ* denotes the sigmoid function. AvgPool is the average pooling method. MaxPool is the max pooling method. $W_{0}$ and $W_{1}$ are the weights of the MLP. $F_{\text{avg}}^{x}$ is the feature map after average pooling. $F_{\max}^{x}$ is the feature map after maximum pooling. x can be taken as c or s. $f^{7\times 7}$ denotes a 7×7 convolution.

**Figure 7:**
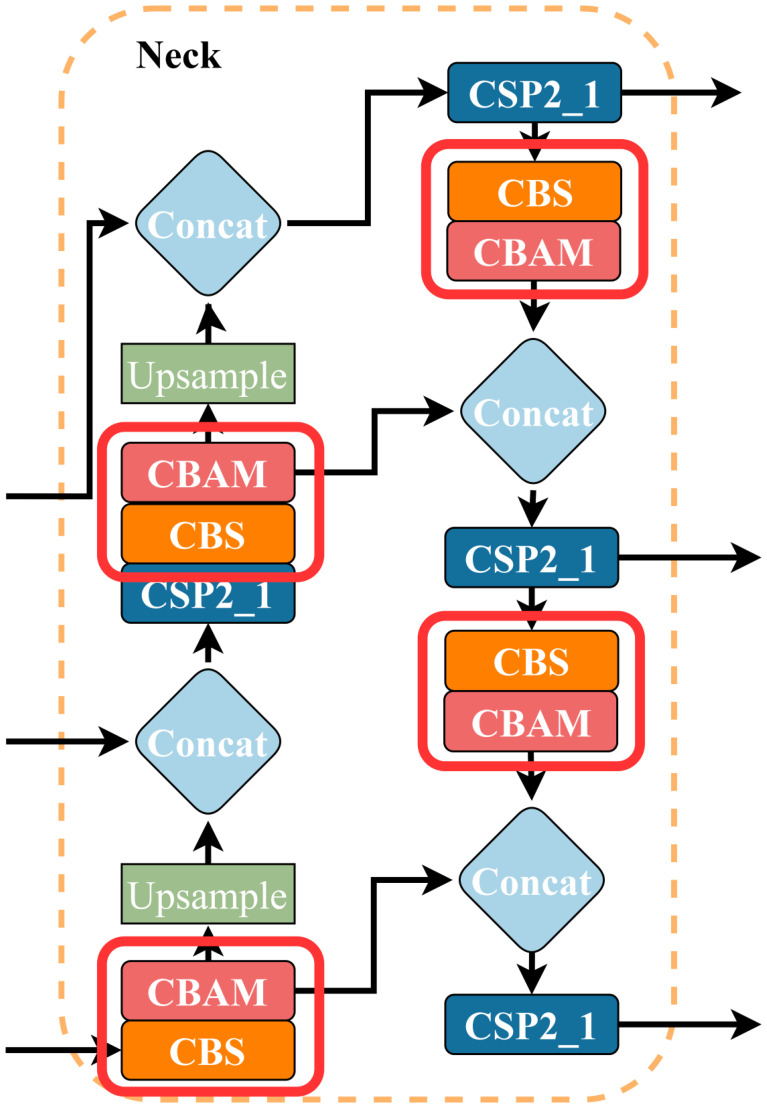
An illustration of 4CBAM’s position in the Neck.

The optimization of the neck layer and the location of the added CBAM modules are shown in **Figure 7**. In this optimization, a total of 4 attention modules are added. The purpose is to use the advantages of the attention modules to improve the model’s ability to work with feature maps and feature information. This improvement is called 4CBAM.

### 3.3 PLDIoU

The PLDIoU (Li et al., 2023) loss is proposed to represent the distance from the predicted box coordinates to the target box coordinates. PLDIoU is calculated from the distance between the prediction box, the target box, and the smallest enclosing box which covers both boxes.

**Figure 8:**
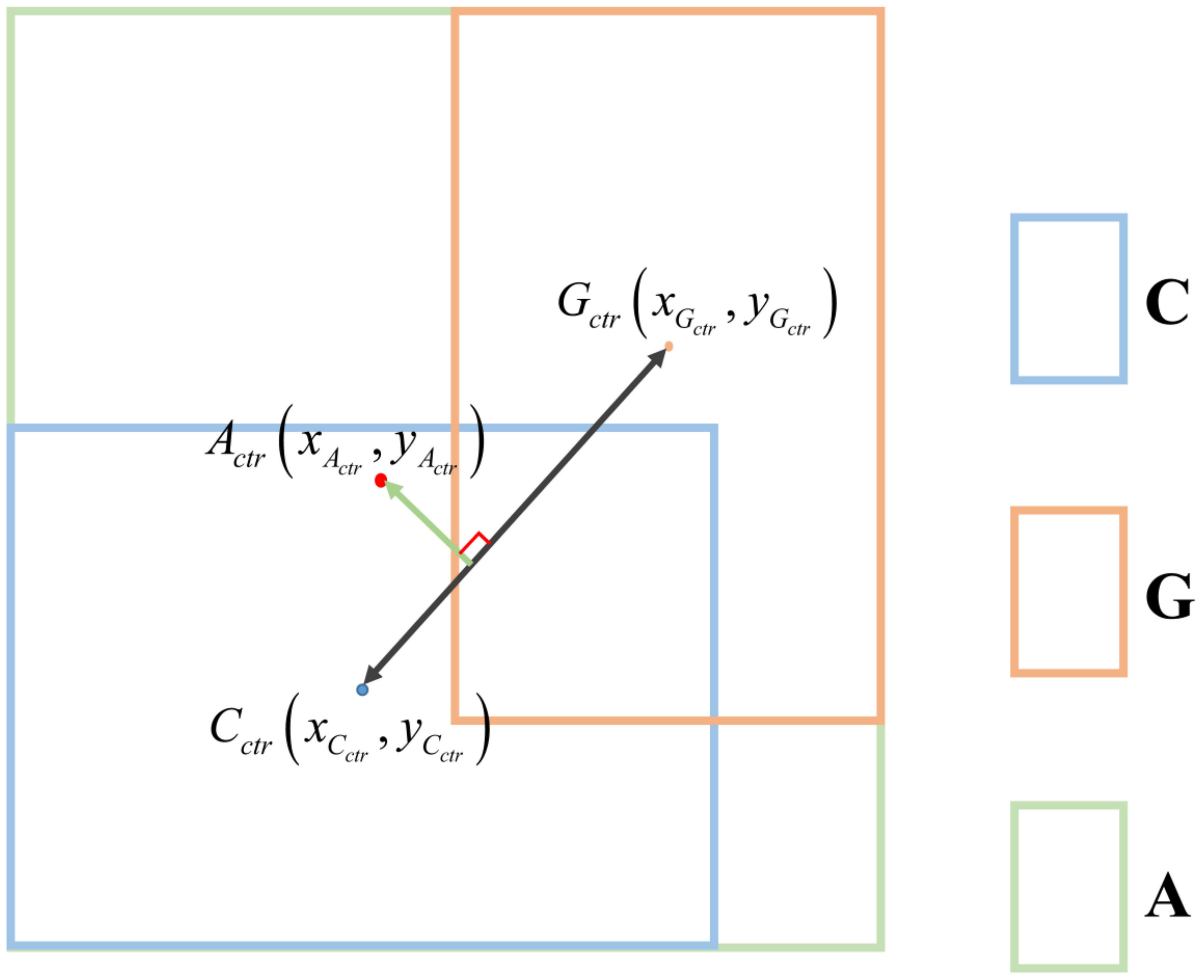
Schematic of *PLDIoU.*.

PLDIoU adds a penalty term $R_{PLDIoU}$ to simplify the calculation of the distance between three boxes by using the point-line distance formula to improve the IoU loss. l is defined as the line from the centroid of the prediction box to the centroid of the target box. After that, the distance d between the center point of the minimum enclosing box and the straight line l is calculated. Finally, we make $R_{PLDIoU}=d^{2}$ . **Figure 8** is a sample PLDIoU diagram. As shown in **Figure 8**, C denotes the prediction box, G denotes the target box, and A is the minimum enclosing box that covers C and G. *C_ctr_* is the center point coordinate of C. *G_ctr_* is the center point coordinate of G. *A_ctr_* denotes the position of the center of A.

The equations for calculating PLDIoU are as follows (Equations 4–11):


(4) $$\alpha =y_{2}-y_{1}$$

(5) $$\beta =x_{2}-x_{1}$$

(6) $$\lambda =x_{2}*y_{1}-x_{1}*y_{2}$$

(7) $$R_{PLDIoU}=\frac{{\left(\alpha x+\beta y+\lambda \right)}^{2}}{\alpha^{2}+\beta^{2}}$$

(8) $$PLDIoU=IoU-\eta R_{PLDIoU}$$

(9) $$L_{PLDIoU}=1-IoU+\eta R_{{\;}_{PLDIoU}}=L_{IoU}+\eta R_{{\;}_{PLDIoU}}$$

(10) $$\begin{array}{l} C\cap G=\max\left(0,\min(C_{x_{2}},G_{x_{2}})-\max(C_{x_{1}},G_{x_{1}})\right)\\ \;\times \max\left(0,\min(C_{y_{2}},G_{y_{2}})-\max(C_{y_{1}},G_{y_{1}})\right) \end{array}$$

(11) $$L_{I\text{oU}}=1-\frac{C\cap G}{(C_{x_{2}}-C_{x_{1}})\times (C_{y_{2}}-C_{y_{1}})+(G_{x_{2}}-G_{x_{1}})\times (G_{y_{2}}-G_{y_{1}})-C\cap G}$$

where $\left(C_{x_{1}},C_{y_{1}}\right)$ denotes the upper left corner coordinates of the prediction box. $\left(C_{x_{2}},C_{y_{2}}\right)$ denotes the lower right corner coordinates of the prediction box. $\left(G_{x_{1}},G_{y_{1}}\right)$ denotes the upper left corner coordinates of the object box. $\left(G_{x_{2}},G_{y_{2}}\right)$ denotes the lower right corner coordinates of the object box.

The result $R_{PLDIoU}$ is obtained by substituting point $C_{ctr}$ , point $G_{ctr}$ and point $A_{ctr}$ into formula (4) to formula (7). Then, the PLDIoU loss function can be calculated by substituting $R_{PLDIoU}$ into formula (8) and formula (9). The η is mainly used to adjust the difference between the two loss function values. From the experiments, it is known that the appropriate value of η is 10.

## 4 Experimental results and analysis

### 4.1 Implementation details

All the model training and validation in this paper were run on GPU image workstations in our lab with the following configurations and versions. Hardware: GPU image workstation with two NVIDIA GeForce RTX 3090 graphics cards; Operating system: Ubuntu 20.04.3; Programming language and open source libraries: Python 3.8, PyTorch v1.10.1, CUDA v11.3, cuDNN v8.0; Hyperparameter settings: During the training process, the initial learning rate of the model is 0.01. As the training is carried out, the learning rate gradually increases and eventually reaches 0.1. Intersection over Union (IoU) loss function coefficient is 0.05. The mosaic algorithm works with a probability of 1. The probability that the mixup method is executed, is 0.5.

### 4.2 Evaluation indicators

P4CN-YOLOv5s is a target detection model. It can be evaluated with indicators of Mean Detection Time (mDT), F_1_-Score, and Mean Average Precision (mAP). These indicators provide a detailed description of the P4CN-YOLOv5s model’s performance with accuracy, speed, and overall performance.

**Figure 9:**
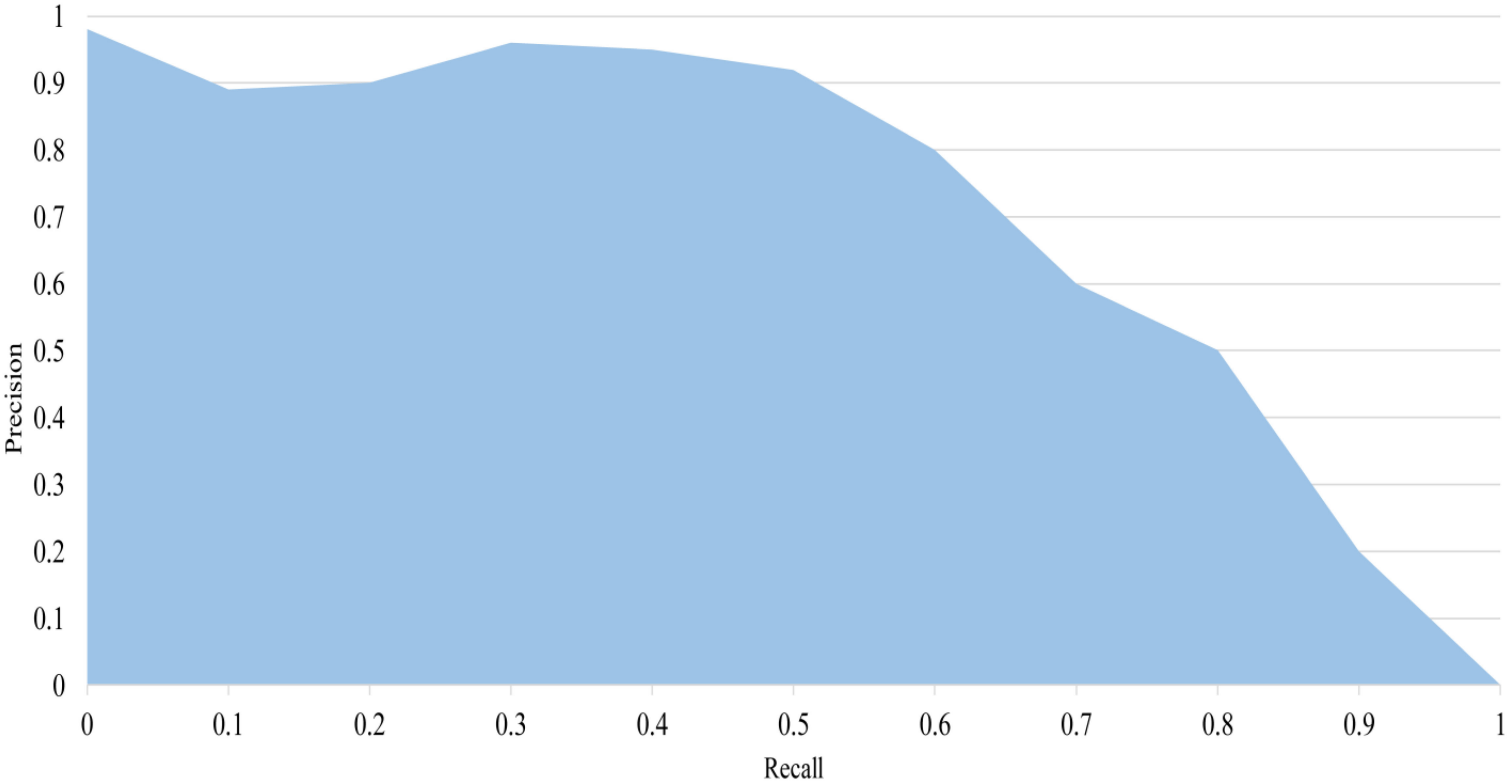
P-R curve. The area of the blue area in the figure is the AP value.

The mAP is a commonly used performance evaluation indicator in target detection. It can be calculated by the arithmetic mean of the Average Precision (AP) of all the categories to be detected. It considers the model’s performance in each category in a comprehensive way, which is a good overall performance evaluation indicator for multi-category target detection. It takes values in the range [0,1]. AP is an indicator of the model which is calculated by the area under the Precision-Recall (P-R) curve. The AP value is very important for evaluating the accuracy of the model in a specific category, and a higher AP value indicates a better performance of the model in that category. The P-R curve is shown in **Figure 9**.

The mDT refers to the average time the model takes from input data to output detection results. It is used to assess the model’s fulfillment of real-time requirements. The F1-score is a combination of Precision and Recall, and their harmonic mean is calculated to balance the precision and omission of the model. The F1 score is a common metric evaluated when a model needs to balance precision and recall. The mathematical formulas are shown below for Precision, Recall, AP, mAP, F1-Score, and mDT (Equations 12–17).


(12) $${\text{P}}_{c}={\frac{TP_{c}}{FP_{c}+TP}}_{c}$$

(13) $${\text{R}}_{c}={\frac{TP_{c}}{FN_{c}+TP}}_{c}$$

(14) $${\text{AP}}_{c}={\sum \int_{0}^{1}P\left(R_{c}\right)dR}_{c}$$

(15) $$mAP=\frac{1}{N}\sum_{i=1}^{N}AP_{i}$$

(16) $$F_{1}\text{-Score=}\frac{2\times P\times R}{P+R}$$

(17) $$\text{mDT=}\frac{1}{M}\sum_{i=1}^{M}t_{i}$$

where *P* is the precision. *R* is the recall. *c* is the current detection category. *TP* is True Positive, *FN* is False Negative, and *FP* is False Positive. *N* is the total number of categories to be detected. *M* is the total number of 1,200 pieces of data to be detected. *t* is the time for detecting an image.

### 4.3 Ablation experiment

The ablation experimental data for the P4CN-YOLOv5s model in **Table 2** can be analyzed to indicate that each ablation model presents optimistic results in the mAP, mDT, and F1 metrics. It shows that modification of the model with different modules can effectively improve the model output.

**Table 2:** Ablation experimental data of P4CN-YOLOv5s.

Models	mAP(%)	mDT(ms)	F1(%)
YOLOv5s	95.27	8.00	93.89
YOLOv5s+PLDIoU	95.50	6.04	94.21
YOLOv5s+4CBAM	95.6	7.25	94.50
YOLOv5s+Mosaic-9	94.63	8.38	93.85
YOLOv5s+Mixup	94.6	6.84	92.98
YOLOv5s+newAnchors	96.04	**5.28**	93.88
YOLOv5s+PLDIoU+4CBAM	96.00	7.90	94.34
YOLOv5s+PLDIoU+4CBAM+Mixup (P4C-YOLOv5s)	96.51	7.70	**95.54**
YOLOv5s+PLDIoU+4CBAM+Mosaic-9+Mixup	96.01	7.21	93.78
YOLOv5s+PLDIoU+4CBAM+ Mosaic-9+Mixup+newAnchors (P4CN-YOLOv5s)	**96.99**	7.20	93.99

Bolding indicates that the indicator is optimal.

From the data of YOLOv5+newAnchors in **Table 2**, the model with the new anchor boxes has improved the mAP by 0.81%, the mDT is reduced to 5.28 ms, and the F1 value is 93.88%. It is determined that the new anchors are more suitable for the model and dataset of this paper.

When PLDIoU, 4CBAM, Mosaic-9, Mixup, and news anchors are integrated into YOLOv5s, i.e., constructing the P4CN-YOLOv5s model, the mAP of P4CN-YOLOv5s is improved by 1.81% to 96.99% relative to the traditional YOLOv5s model. Meanwhile, the mDT and F1 of P4CN-YOLOv5s are 7.2 ms and 93.99%, respectively, which are both better than the traditional YOLOv5s model.

**Figure 10:**
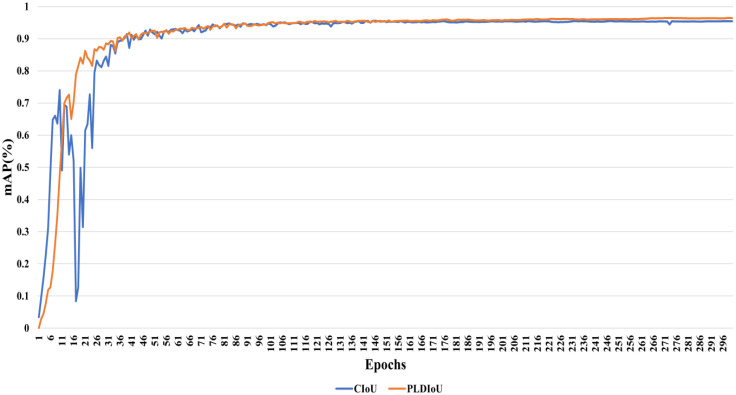
The mAP comparison between the PLDIoU and the CIoU.

Furthermore, the iterative values of mAP for PLDIoU loss and CIoU loss during training are shown in **Figure 10**. Its analysis shows that the PLDIoU model is more stable than the CIoU model in the whole training. Roughly 101 epochs later, the mAP value of the PLDIoU model surpasses the mAP value of the CIoU model.

**Figure 11:**
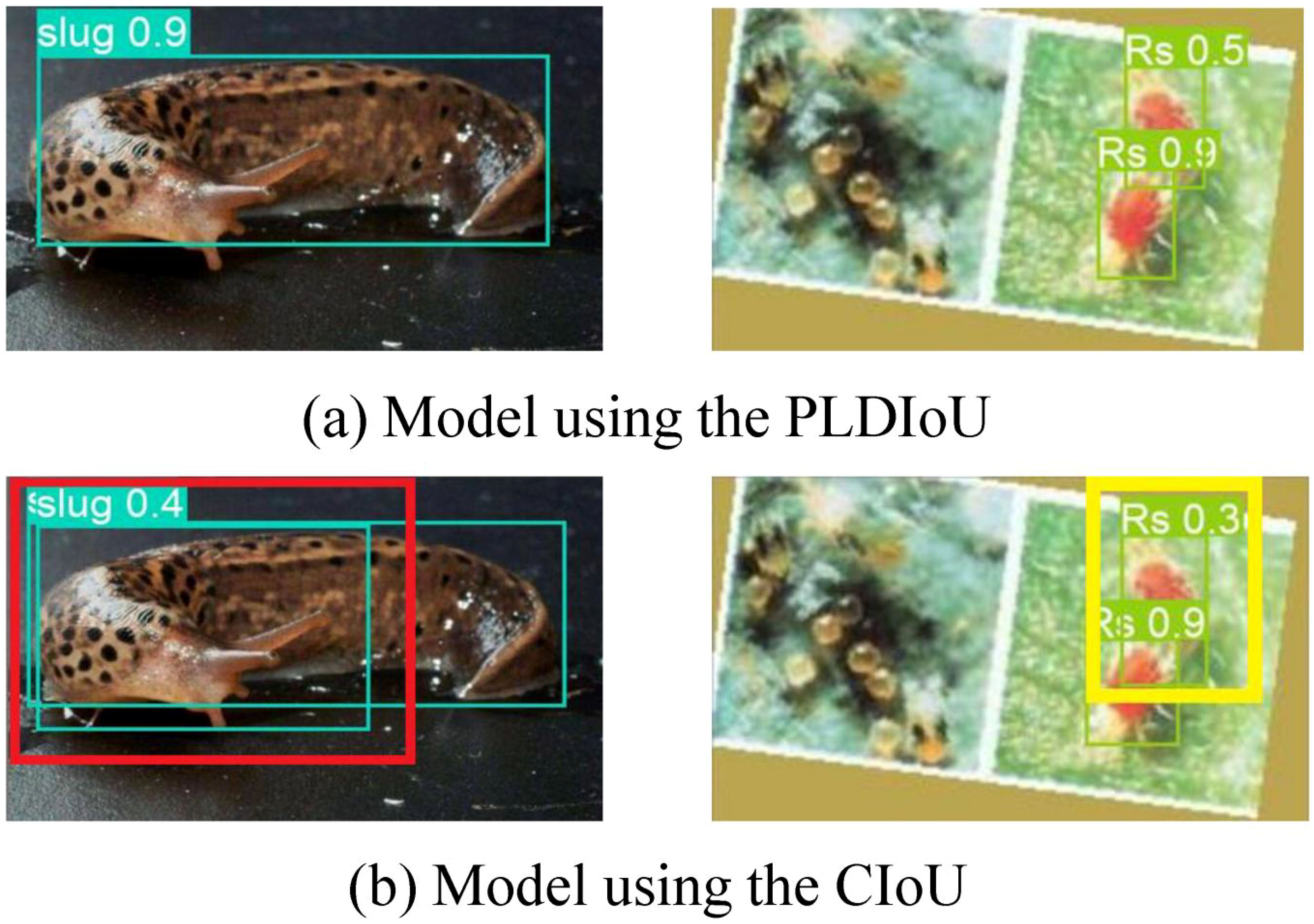
Detection comparison of models using different IoU **(a)** model using the CIoU **(b)** model using the PLDIoU.

**Figure 11** displays the outcome of the model using the PLDIoU function and the model using the CIoU function for passion fruit pest detection. **Figure 11a** shows the outcome of the model using PLDIoU function. The red and yellow boxes in **Figure 11b** show that the CIoU model has the problems of multiple boxes and oversized boxes. From **Figures 10**, **11**, and the data of YOLOv5+PLDIoU in **Table 2**, it is clear that PLDIoU performs stably and helps to speed up the convergence. Meanwhile, PLDIoU not only resolves the multi-box and oversized box issues but also improves the accuracy of the model.

**Figure 12:**
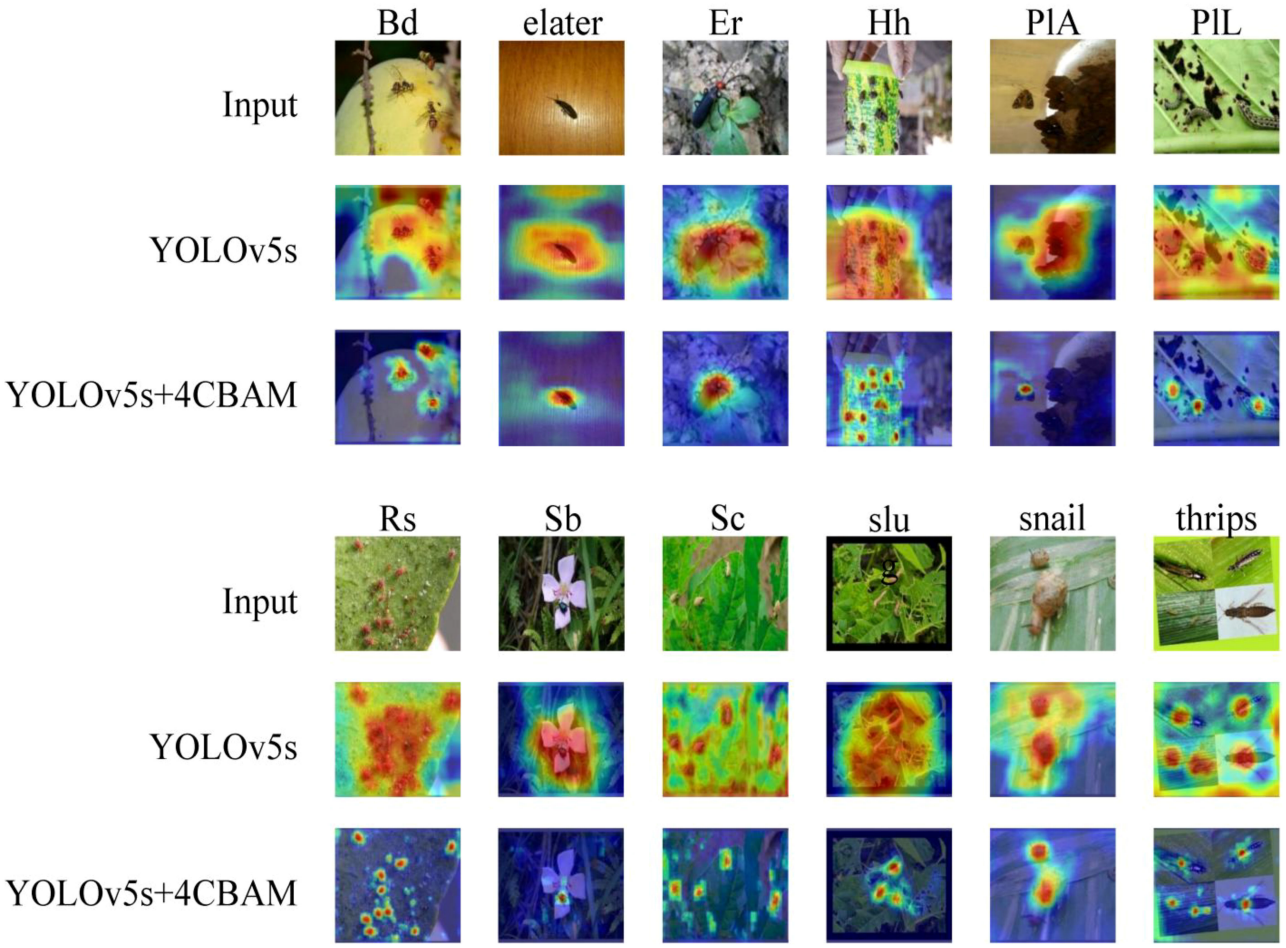
Shows the output of the Grad-CAM visualization.

As a common visualization method in deep learning, Grad-CAM (Zhang et al., 2018) highlights the interest regions by using gradient computation. **Figure 12** presents a comparison of the visualization results of the 4CBAM integrated network (YOLOv5s+4CBAM) and YOLOv5s. It shows that the 4CBAM integrated network supports the model to improve the coverage of target objects and output better and more accurate results. It reduces the attention to unimportant information. 4CBAM is effective.

### 4.4 Comparison with other algorithms

A comparison is made between the P4CN-YOLOv5s model and commonly used target detection models from which we can objectively validate the proposed P4CN-YOLOv5s model. The detailed results are summarized in **Tables 3**, **4**, and **Table 5**.

**Table 3:** The comparison results between P4CN-YOLOv5s and mainstream algorithms (Part 1).

Models	Input Size	mAP(%)	mDT(%)	$F_{1}\text{-Score}$ (%)
SSD	300×300	81.71	23.9	72.52
Faster R-CNN	600×600	88.38	25	73.42
YOLOv3	416×416	93.29	18.7	90.17
YOLOv4	416×416	75.71	11.7	68.92
YOLOv5s	640×640	95.27	8	93.89
P4C-YOLOv5s	640×640	96.51	7.7	**95.54**
YOLOv7	640×640	95.38	7.5	93.76
P4CN-YOLOv5s	640×640	**96.99**	**7.2**	93.99

Bolding indicates that the indicator is optimal.

**Table 4:** The comparison results between P4CN-YOLOv5s and mainstream algorithms (Part 2).

Models	AP(%)					
	Bd	Elater	Er	Hh	PlA	PlL
SSD	75	90.6	80.5	90.5	90.3	86.6
Faster R-CNN	81.91	96.72	90.42	97.84	98.59	93.32
YOLOv3	90.99	98.27	94.44	98.66	99.03	98.75
YOLOv4	68.76	69.57	69.44	92.16	97.72	85.31
YOLOv5s	93.6	99.3	95.1	99.2	98.5	96.4
P4C-YOLOv5s	90.2	99.2	97.3	99	99.5	98.2
YOLOv7	90.6	99.2	95.8	99	98.5	96.8
P4CN-YOLOv5s	92.7	99.3	96.3	99.1	98.4	97.4

**Table 5:** The comparison results between P4CN-YOLOv5s and mainstream algorithms (Part 3).

Models	AP(%)					
	Rs	Sb	Sc	Slug	Snail	Thrips
SSD	74.1	82.7	83	86.2	83.1	57.8
Faster R-CNN	80.64	87.11	92.33	90.19	87.86	63.57
YOLOv3	93.44	88.44	95.6	93.45	92.42	76.02
YOLOv4	88.85	69.88	66.02	71.2	70.91	58.67
YOLOv5s	94	92.9	98.5	94.2	95.1	86.4
P4C-YOLOv5s	97	95.3	96.3	94.6	94.7	96.8
YOLOv7	95.4	93	96.2	94.8	95.6	96.5
P4CN-YOLOv5s	96.5	95.2	98	97.8	96	97.2

The mAP value of the P4CN-YOLOv5s model is 96.99%, which is 18.7%, 9.74%, 3.97%, 28.11%, 1.81%, 1.61% and 0.5% better than SSD (Liu et al., 2016), Faster R-CNN (Ren et al., 2017), YOLOv3 (Redmon and Farhadi, 2018), YOLOv4 (Bochkovskiy et al., 2020), YOLOv5s (Jocher et al., [[NoYear]]), YOLOv7 (Wang et al., 2023)and P4C-YOLOv5s (Li et al., 2023), respectively. The mDT value in the P4CN-YOLOv5s model is 7.2 milliseconds, which is the shortest detection time in the comparison experiment and meets the real-time requirement. From the comparative experimental data, it is clear that the P4CN-YOLOv5s model has improved in mAP, mDT and F1, which meets the accuracy and real-time requirements. The experiments prove that the P4CN-YOLOv5s model is effective in passion fruit pest detection.

**Figure 13:**
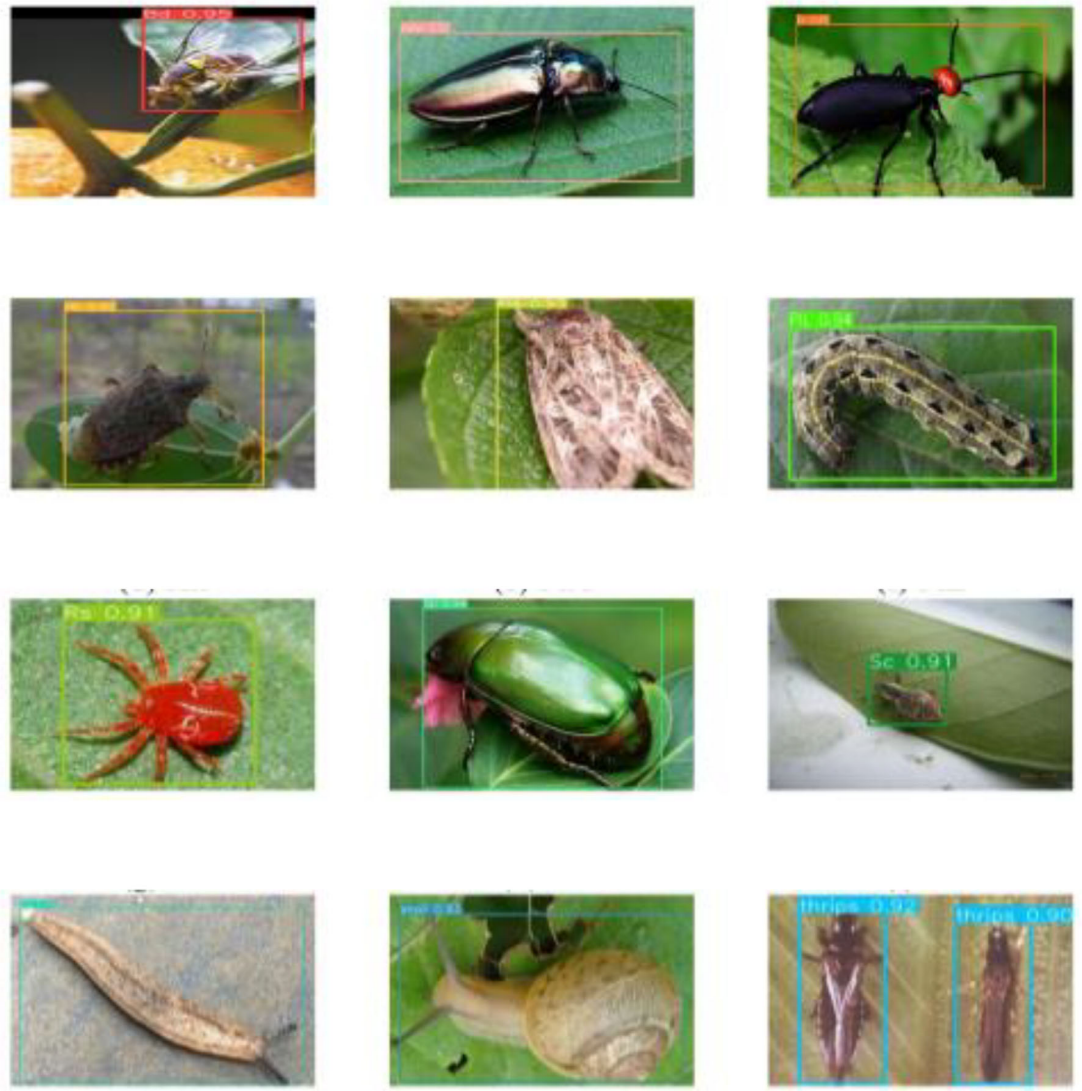
The results of P4CN-YOLOv5s model detection.

The trained P4CN-YOLOv5s model is validated with the test dataset, and the results are shown in **Figure 13**. It is observed that the P4CN-YOLOv5s model can accurately identify and locate the object pests.

## 5 Conclusion

This paper proposed a P4CN-YOLOv5s model for passion fruit pest detection to improve the accuracy and speed of passion fruit pest detection. The Mixup algorithm and Mosaic-9 algorithm are added to the input layer to improve the dataset complexity and model robustness. Then, four CBAM modules are used on the neck layer to make the model focus on the object and improve the accuracy. In addition, the new PLDIoU loss function is used in the prediction layer to reduce the false detection rate and speed up the localization. Finally, the model’s anchor boxes are readjusted. The experimental results show that the P4CN-YOLOv5s model has a mAP value of 96.99%, an mDT value of 7.2 ms, and an F1 value of 93.99%, which meets the requirements of accuracy and speed. Although the P4CN-YOLOv5 model’s accuracy and speed have been improved, it suffers from missed detection and decreased accuracy in dark environments. In addition, no comparative experiments were conducted with loss functions like CIoU or DIoU. Future work will focus on incorporating low-light image enhancement techniques, such as image denoising and infrared sensing, to improve model performance in challenging lighting conditions. Additionally, we plan to conduct a thorough evaluation comparing PLDIoU with these advanced loss functions to better understand their respective impacts on detection accuracy and efficiency, and to further refine the model’s performance. These efforts will enhance the model’s robustness and generalizability, ensuring its effectiveness in diverse real-world scenarios.

## Data Availability

The original contributions presented in the study are included in the article/supplementary material. Further inquiries can be directed to the corresponding author.
